# The Epidemiology of Unintentional and Violence-Related Injury Morbidity and Mortality among Children and Adolescents in the United States

**DOI:** 10.3390/ijerph15040616

**Published:** 2018-03-28

**Authors:** Michael F. Ballesteros, Dionne D. Williams, Karin A. Mack, Thomas R. Simon, David A. Sleet

**Affiliations:** 1 Division of Analysis, Research and Practice Integration, National Center for Injury Prevention and Control, Centers for Disease Control and Prevention, Atlanta, GA 30341, USA; duw0@cdc.gov (D.D.W.); kim9@cdc.gov (K.A.M.); 2 Division of Violence Prevention, National Center for Injury Prevention and Control, Centers for Disease Control and Prevention, Atlanta, GA 30341, USA; tgs9@cdc.gov; 3 The Bizzell Group, LLC, Lanham, MD 20706, USA; davidasleet@gmail.com; 4 Division of Unintentional Injury Prevention, National Center for Injury Prevention and Control, Centers for Disease Control and Prevention, Atlanta, GA 30341, USA

**Keywords:** injury, homicide, suicide, children, adolescents, epidemiology

## Abstract

Injuries and violence among young people have a substantial emotional, physical, and economic toll on society. Understanding the epidemiology of this public health problem can guide prevention efforts, help identify and reduce risk factors, and promote protective factors. We examined fatal and nonfatal unintentional injuries, injuries intentionally inflicted by other (i.e., assaults and homicides) among children ages 0–19, and intentionally self-inflicted injuries (i.e., self-harm and suicides) among children ages 10–19. We accessed deaths (1999–2015) and visits to emergency departments (2001–2015) for these age groups through the Centers for Disease Control and Prevention’s (CDC) Web-based Injury Statistics Query and Reporting System (WISQARS), and examined trends and differences by age, sex, race/ethnicity, rural/urban status, and injury mechanism. Almost 13,000 children and adolescents age 0–19 years died in 2015 from injury and violence compared to over 17,000 in 1999. While the overall number of deaths has decreased over time, there were increases in death rates among certain age groups for some categories of unintentional injury and for suicides. The leading causes of injury varied by age group. Our results indicate that efforts to reduce injuries to children and adolescents should consider cause, intent, age, sex, race, and regional factors to assure that prevention resources are directed at those at greatest risk.

## 1. Introduction

Injuries and violence among young people continue to have a substantial emotional, physical, and economic toll on society. On average, a child dies every hour in the United States from an injury or violent act, and over 30% of all deaths among children age 1–19 years are from injuries or violence [1,2]. Another 22,200 children on average are seen every day in U.S. emergency departments (ED) seeking treatment for nonfatal injuries [1,2]. Injuries can be intentional, such as from an assault, homicide, or suicide/self-harm, or they can be unintentional, such as from motor vehicle crashes, drowning, poisoning, fires/burns, falls, or suffocation. Injuries can have lasting consequences and result in a substantial economic burden on society. Each year, injuries to U.S. children 0–19 years of age result in an estimated $94 billion in lifetime medical and work-loss costs [1]. 

Understanding epidemiology is critical to injury control as it can provide a picture of the public health and economic burdens that can guide prevention efforts, help identify and reduce risk factors, and increase protective factors. The purpose of this study is to use recent U.S. data to examine in detail the epidemiology of injuries among children ages 0–19 years by age, sex, race/ethnicity, rural/urban status, and injury mechanism. We specifically focus on trends and differences by injury intent and age group for both fatal and non-fatal injury rates. 

## 2. Methods

We examined data on fatal and non-fatal injuries among children and teens aged 0–19 years using two large data systems. All data were accessed using the Centers for Disease Control and Prevention’s (CDC) Web-based Injury Statistics Query and Reporting System (WISQARS), which is a free, interactive, online database that provides information on fatal and nonfatal injuries and cost of injuries from a variety of trusted sources [1,3]. Data are publically available and provide no personal identifiable information. Institutional Review Board (IRB) approval was not needed for this study.

### 2.1. Mortality

Data on deaths due to injuries and violence came from 1999–2015 WISQARS fatal reports, which uses the National Vital Statistics System (NVSS). NVSS receives death certificate data from 50 states and the District of Columbia. Injury deaths were defined as those with an underlying cause of death classified by external cause of injury codes (*International Classification of Diseases, 10th Revision* ICD-10; https://www.cdc.gov/injury/wisqars/fatal_help/data_sources.html). Unintentional injury ICD-10 codes used were V01–X59 or Y85–Y86. Homicide and legal intervention (hereafter, referred to as homicides) deaths included ICD-10 codes X85-Y09, Y87.1, Y35, Y89.0, or *U01-*U02 (terrorism); and suicide deaths included codes X60–X84, Y87.0, or *U03 (terrorism). Unintentional injury did not include iatrogenic injuries (Y40–Y84) and intentional injury did not include operations of war (Y36). The number of suicide deaths and rates were calculated only for youth 10 years of age and older. Children aged <10 years were excluded because suicidal intent is often not attributed to young children. These detailed codes for injury deaths are expressed in mechanism (cause) groupings in WISQARS (e.g., motor vehicle traffic (MV) occupants, poisonings, fires/burns, falls, and suffocations) using the external cause-of-injury mortality matrix [4]. Deaths for individuals coded as unspecified MV traffic road users were included with MV occupant deaths [5]. While the mechanism of homicide and suicide deaths (e.g., firearm, poisoning) can be determined on WISQARS, they are combined in this analysis because of low counts in some data cells. 

Metropolitan (‘metro’) or non-metropolitan (‘non-metro’) categories were assigned to deaths based on the National Center for Health Statistics’ (NCHS) six-level county-based urbanization classifications for 2013 [6]. The ‘metro’ classification was defined by collapsing urbanization codes for large central metro, large fringe metro, medium metro, and small metro counties; and the ‘non-metro’ classification included micropolitan and non-core counties. This paper highlights results from the collapsed two-level metro/non-metro classification based on the 2013 urbanization classification scheme that was applied to the 1999–2015 data. We examined death rates by age group, sex, race/ethnicity, injury mechanism, metro/non-metro status, state, and year.

### 2.2. Morbidity

Data on individuals treated in U.S. EDs for non-fatal injuries came from the 2001–2015 National Electronic Injury Surveillance System-All Injury Program (NEISS-AIP). The NEISS is an ongoing surveillance system that monitors consumer product-related injuries treated in U.S. hospital EDs. The U.S. Consumer Product Safety Commission maintains and operates NEISS, which currently includes 100 hospital emergency departments (ED) that represent a stratified probability sample of all U.S. and U.S. territory hospitals that have at least six beds and provide 24-hour emergency services. NEISS-AIP is a subsample of 66 of the 100 NEISS hospitals. NEISS-AIP tracks all injuries seen in EDs, whether or not they are associated with consumer products. Data from these cases are weighted by the inverse of the probability of selection to provide national estimates. Non-fatal injuries were examined by manner (unintentional, intentionally inflicted by another—including sexual assault and legal intervention—hereafter ‘assault’—and self-harm), age group and mechanism. All definitions of causes are provided on the WISQARS website [1], but note that “struck by/against or crushed” is defined as an injury resulting from being struck by (hit) or crushed by a human, animal, or inanimate object or force other than a vehicle or machinery.

Fatal and non-fatal rates were calculated using the U.S. Census population totals with bridged race categories [7]. 

## 3. Results

In 2015, there were 12,977 injury deaths among individuals 0–19 years of age, with an overall crude death rate of 16.2 per 100,000 population. In contrast, there were 17,632 injury deaths in 1999. In 2015, 61% of deaths were unintentional, 20% were homicides, and 19% were suicides. Males accounted for 69% of the deaths and had an overall crude death rate over two times greater than females (21.3 versus 10.0). Infants (less than one year old) (39.1) and youth 15–19 (36.0) years old had the highest injury crude death rates (Table 1).

Additionally, in 2015, we estimate there were over 8.1 million children 0–19 years of age treated in a U.S. ED for a non-fatal injury (versus over 10.3 million in 1999), with 95% of these visits for unintentional injuries compared with 4% for assaults and 2% for self-harm injuries. The highest estimated non-fatal injury rates were among those ages 1–4 years (11,196 per 100,000 population) and ages 15–19 years (11,952 per 100,000). Estimated rates of ED visits (9352 per 100,000) for unintentional injury were almost 20 times higher than for intentional injury (371 per 100,000). Males accounted for 57% of the non-fatally injured patients (Table 1). 

### 3.1. Leading Causes of Injury Mortality and Morbidity

#### 3.1.1. Mortality

For infants under the age of one, unintentional suffocation was the leading cause of injury death, with over four times the number of deaths as the second leading cause of injury death, homicide (Table 2). Unintentional drowning was the leading cause of injury death for children 1–4 years old, and motor vehicle traffic death was the leading cause of injury death for children 5 years and older. Homicides were either the second or the third leading cause of injury death in all age groups, while suicides ranked second for those 10–14 and 15–19 years old. For all age groups, the leading injury mechanisms for homicide were firearm, cut/pierce, and suffocation; and the leading mechanisms for suicide were suffocation, firearm, and poisoning (data not shown).

#### 3.1.2. Morbidity

Among children younger than 15 years, unintentional falls were the leading cause of ED visits for injury (Table 3). Unintentionally being struck by/against was the leading cause of ED visits for those 15–19 years, and the second leading cause for all other age groups. We estimate almost 250,000 youth 10–19 years of age were seen in an ED for an assault (the most common injury mechanism being struck by/against, sexual assault, cut/pierce, and firearm); almost 130,000 in the same age group were seen for self-harm injuries (poisoning, cut/pierce, and other specified mechanisms).

### 3.2. Trends in Mortality and Morbidity

#### 3.2.1. Mortality

**Figure 1:**
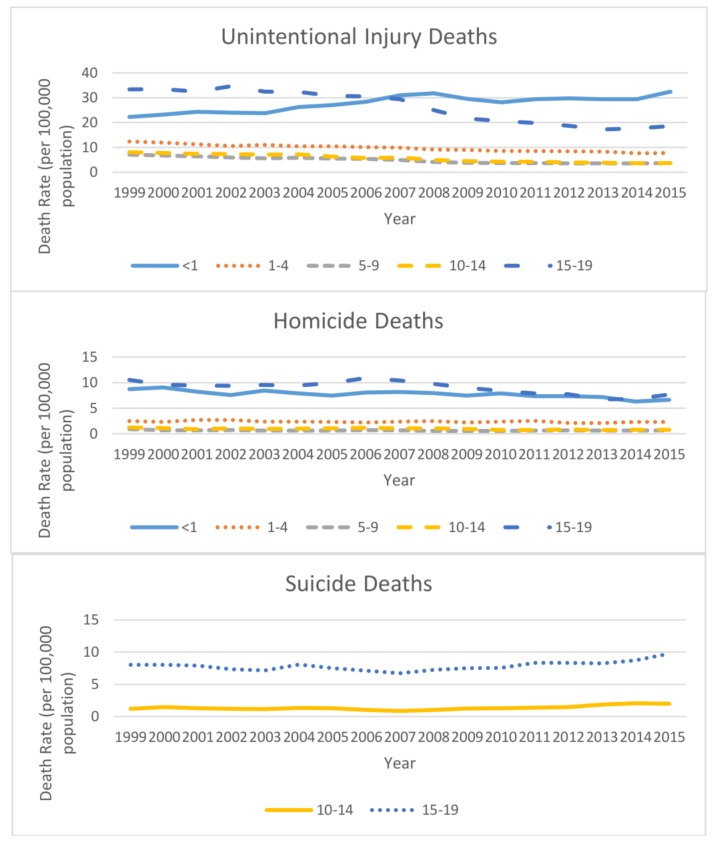
Injury and violence-related death rates (per 100,000 population) among youth aged 0–19 years by intent and age group, National Vital Statistics System, United States, 1999–2015.

Individuals younger than 1 and 15–19 years of age had the highest unintentional injury death rates between 1999 and 2015 (Figure 1) with the unintentional injury death rate among infants increasing approximately 46% (22.3 in 1999 to 32.5% in 2015) and rate among 15–19 age group decreasing 44% (33.3 in 1999 to 18.6 in 2015). The rates for all other age groups decreased between 37 and 54%. Those aged 1–4, 5–9, and 10–14 years had lower rates each year than infants or those aged 15–19 years. The lowest death rates were found among children aged 5–9 years, ranging from 3.56 to 7.08 over this time period. Notably, the death rates of infants (31.0) surpassed that of adolescents aged 15–19 years (29.4) beginning in 2007. 

Homicide death rates also were highest among those <1 (ranging from 6.3 to 9.1) and 15–19 (ranging from 6.7 to 10.9) years of age between 1999 and 2015 (Figure 1). Homicide death rates decreased over the 17-year study period for all age groups. Suicide deaths were reported only for the two older age groups. Suicide death rates increased for both age groups between 2007 and 2015, with the increases from 2007 and 2015 being 130% and 46% for the 10–14 and 15–19 year olds, respectively (Figure 1).

#### 3.2.2. Morbidity

**Figure 2:**
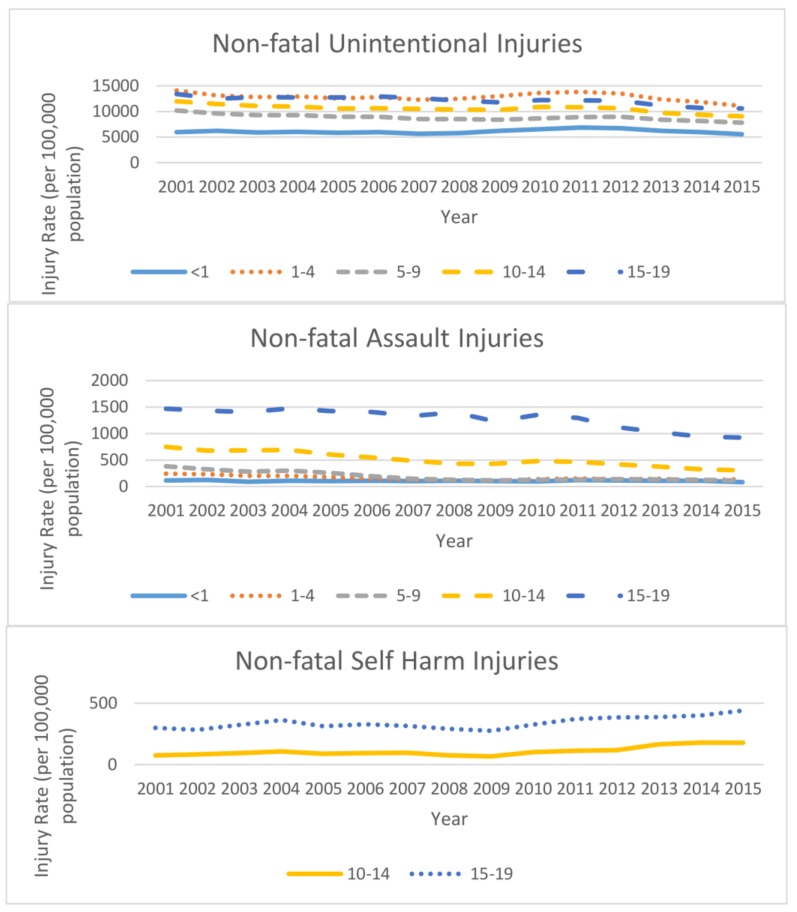
Non-fatal injury rates (per 100,000 population) among youth aged 0–19 years by intent, age group, and year, National Electronic Injury Surveillance System-All Injury Program, United States, 2001–2015.

Individuals aged 1–4 and 15–19 years had the highest non-fatal unintentional injury rates (Figure 2). The non-fatal unintentional injury rates decreased in all age groups, with the age-adjusted rate for all ages combined decreasing 22.0% from an estimated 11,988 per 100,000 in 2001 to 9352 in 2015. Non-fatal injury rates for assaults among 15–19 year olds (ranging from 1471 in 2001 to 923 in 2015) were over twice the rates of the other age groups. The non-fatal injury rate for assaults among those aged 0–19 years was estimated to be 712 in 2001 and 372 in 2015, a 47.8% decrease. Rates for non-fatal self-harm increased by over 130% among 10–14 year olds (76 in 2001, 178 in 2015), and increased by 47% among 15–19 year olds (299 in 2001, 440 in 2015) (Figure 2). 

### 3.3. Injury Mortality by States and Urban Status

#### 3.3.1. States

**Figure 3:**
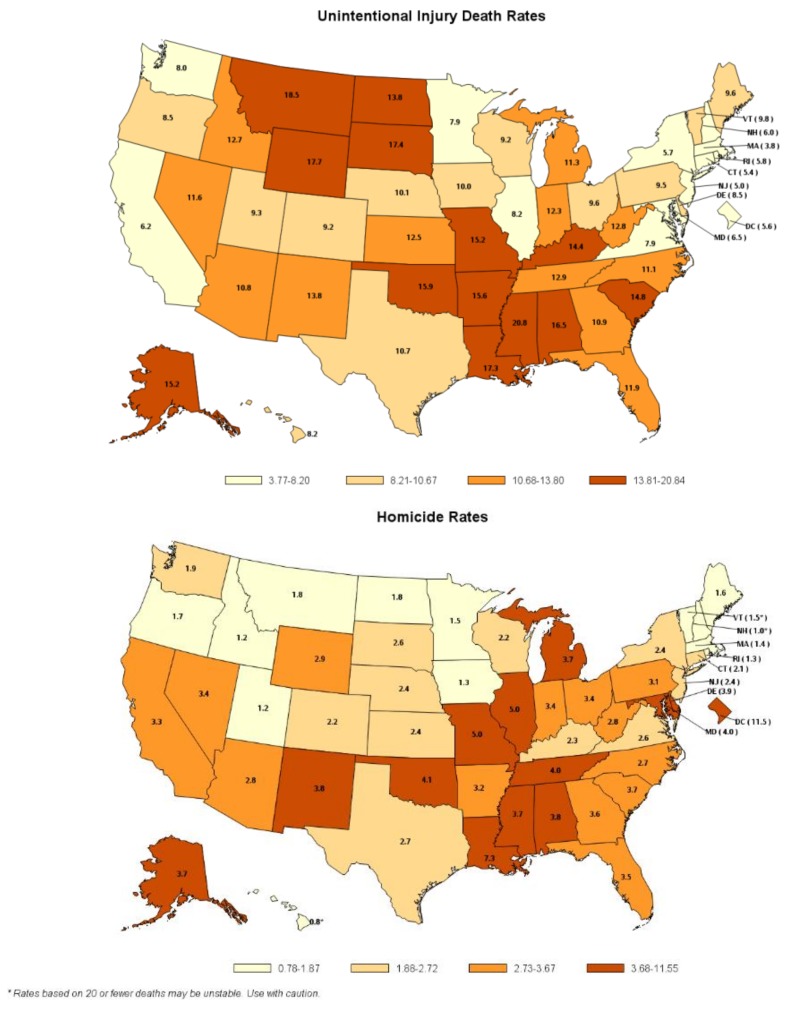

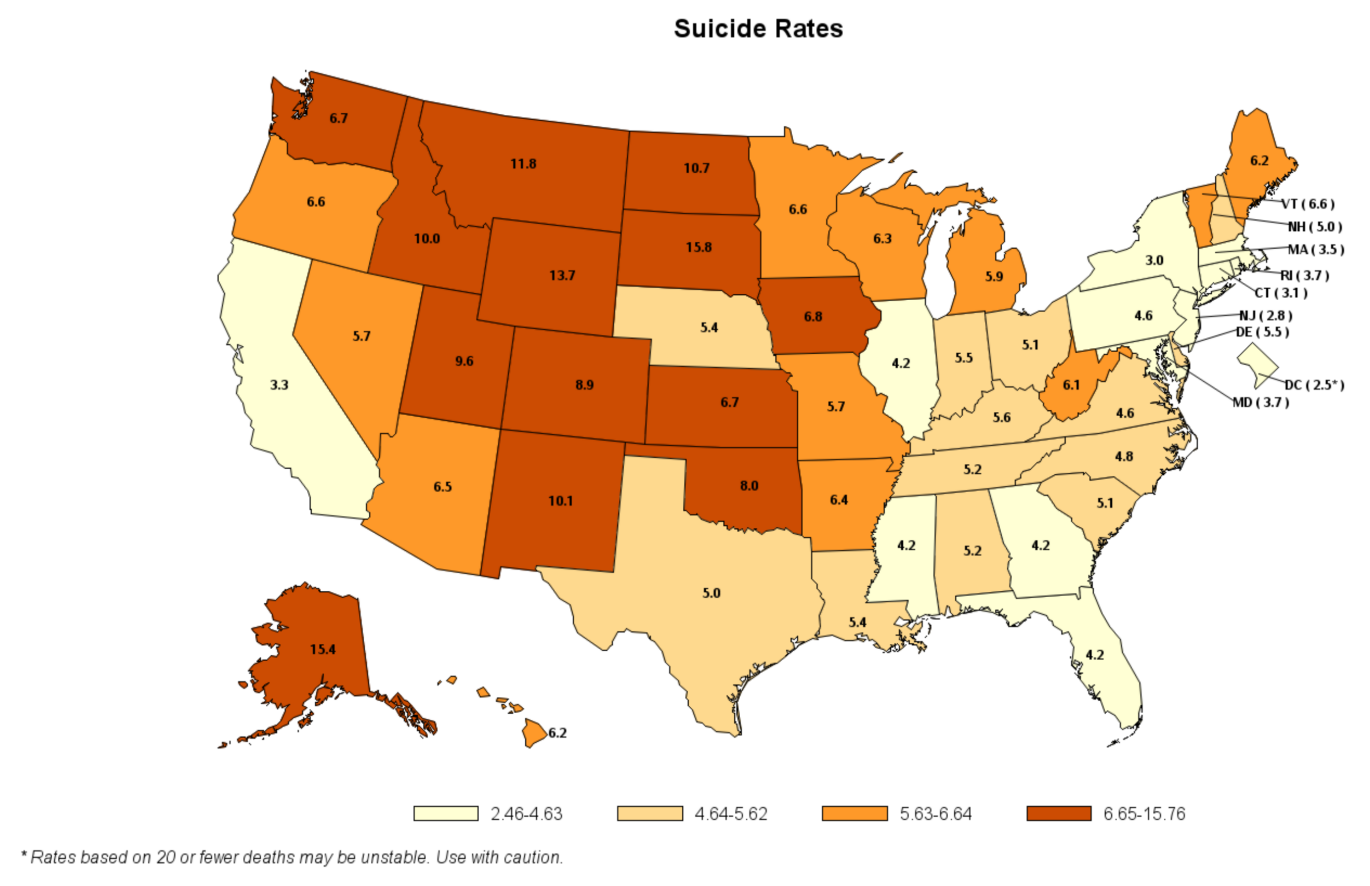
Injury and violence related death rates (per 100,000 population) among youth aged 0–19 years by intent and state, National Vital Statistics System, United States, 2009–2015. Note: data for Suicides is only for 10–19 year olds.

Age-adjusted death rates (2009–2015 combined) by intent varied greatly across states and regions of the United States (Figure 3). Unintentional injury death rates generally were high in the southeast and low in New England and the Mideast. States with rates in the highest quartile included AL, AK, AR, KY, LA, MO, MS, MT, ND, OK, SC, SD, and WY. Homicide death rates were lowest in New England and states along the western northern border (e.g., ID, MN, MT, ND, OR) of the United States and highest in the middle of the country (e.g., AL, IL, OK, LA, MI, MO, MS, NM, TN). Suicide death rates were high in the Rocky Mountains (e.g., CO, ID, MT, UT, WY) and lower in the Southeast (e.g., GA, FL, MS). 

#### 3.3.2. Urban Status

Age-adjusted unintentional injury death rates in metro areas were 13.7 in 1999 and 8.5 in 2015, while rates in non-metro areas were 25.9 in 1999 and 16.7 in 2015. Unintentional injury death rates among youth aged 0–19 years in metro areas were 47.3% lower than non-metro areas in 1999 and 49.2% lower in 2015. Age-adjusted homicide death rates in metro areas were 4.4 in 1999 and 3.2 in 2015, while rates in non-metro areas were approximately 2.5 in both 1999 and 2015, resulting in a rate decrease of 28.4% in metro areas from 1999 to 2015 compared with only a 2.0% decrease in non-metro areas. Homicide rates were 76.5% higher in metro versus non-metro areas in 1999 versus 28.9% higher in 2015. Age-adjusted suicide death rates (for 10–19 year olds) in metro areas were 4.3 in 1999 and 5.4 in 2015, while rates in non-metro areas were 6.1 in 1999 and 8.4 in 2015. This resulted in suicide rate increases over this time period of 25.8% in metro areas and 39.3% in non-metro areas.

#### 3.3.3. Race and Ethnicity

**Figure 4:**
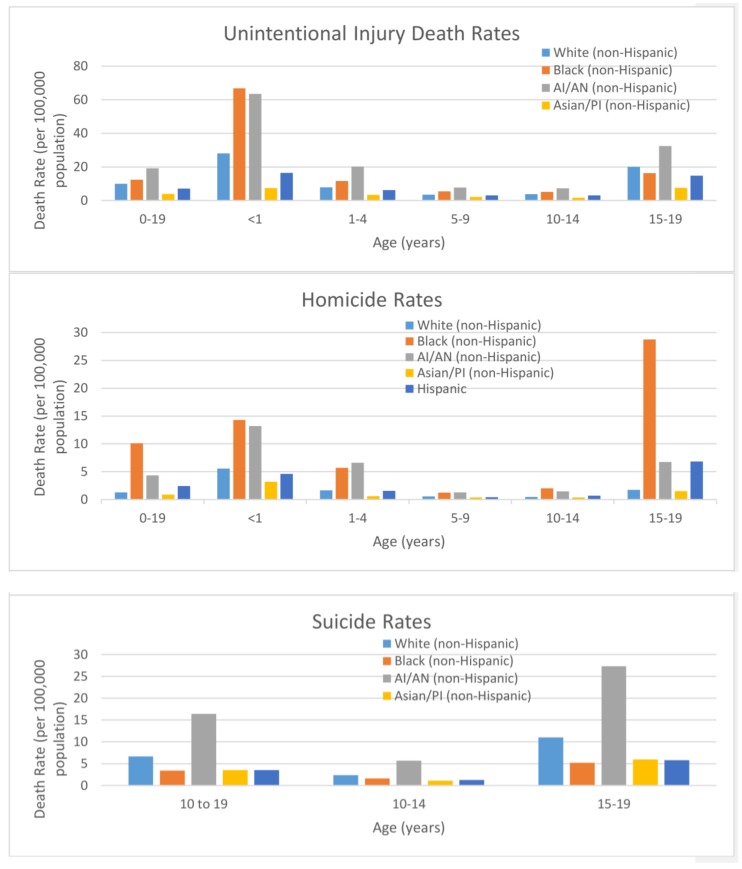
Injury and violence related death rates (per 100,000 population) among youth aged 0–19 years by intent and race, National Vital Statistics System, United States, 2013–2015.

Unintentional injury death rates from 2013–2015 were highest among American Indian and Alaskan Native peoples (AI/AN), with the highest rates among AI/AN (63.6) and Black (66.8) infants, and AI/AN 15–19 year olds (32.5) (Figure 4). Overall, homicide rates were highest among Blacks (10.1 for all ages combined), with Blacks 15–19 years old having homicide rates over twice (28.8) any other age and race category. Suicide rates were highest among AI/AN (5.7 for all ages combined) in our study population, with AI/AN 15–19 year olds (27.3) having the highest death rates.

### 3.4. Injury Death Rates by Sex, Single Year of Age, Intent, and Mechanism

**Figure 5:**
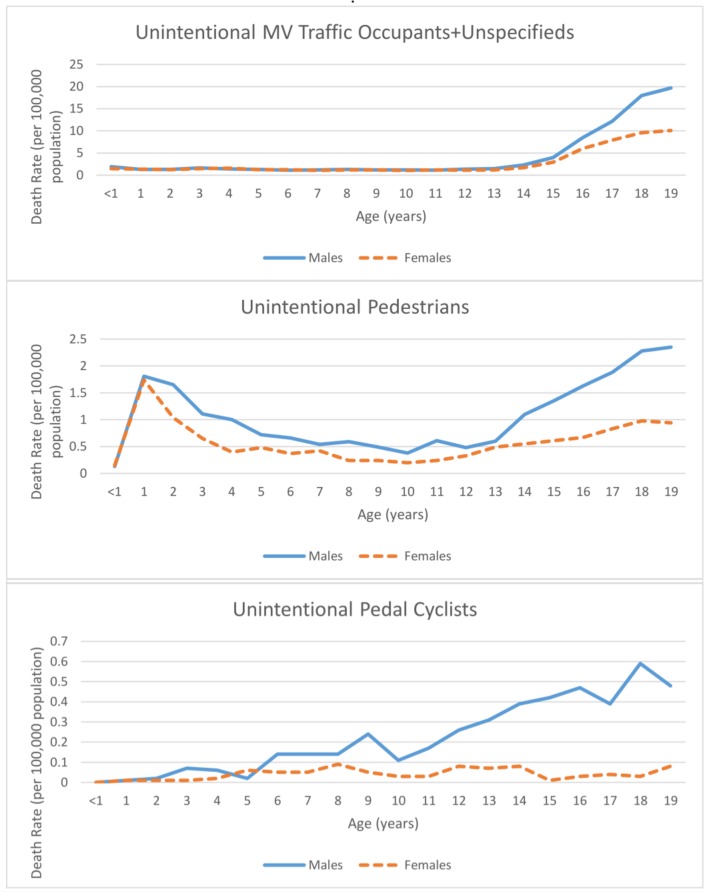

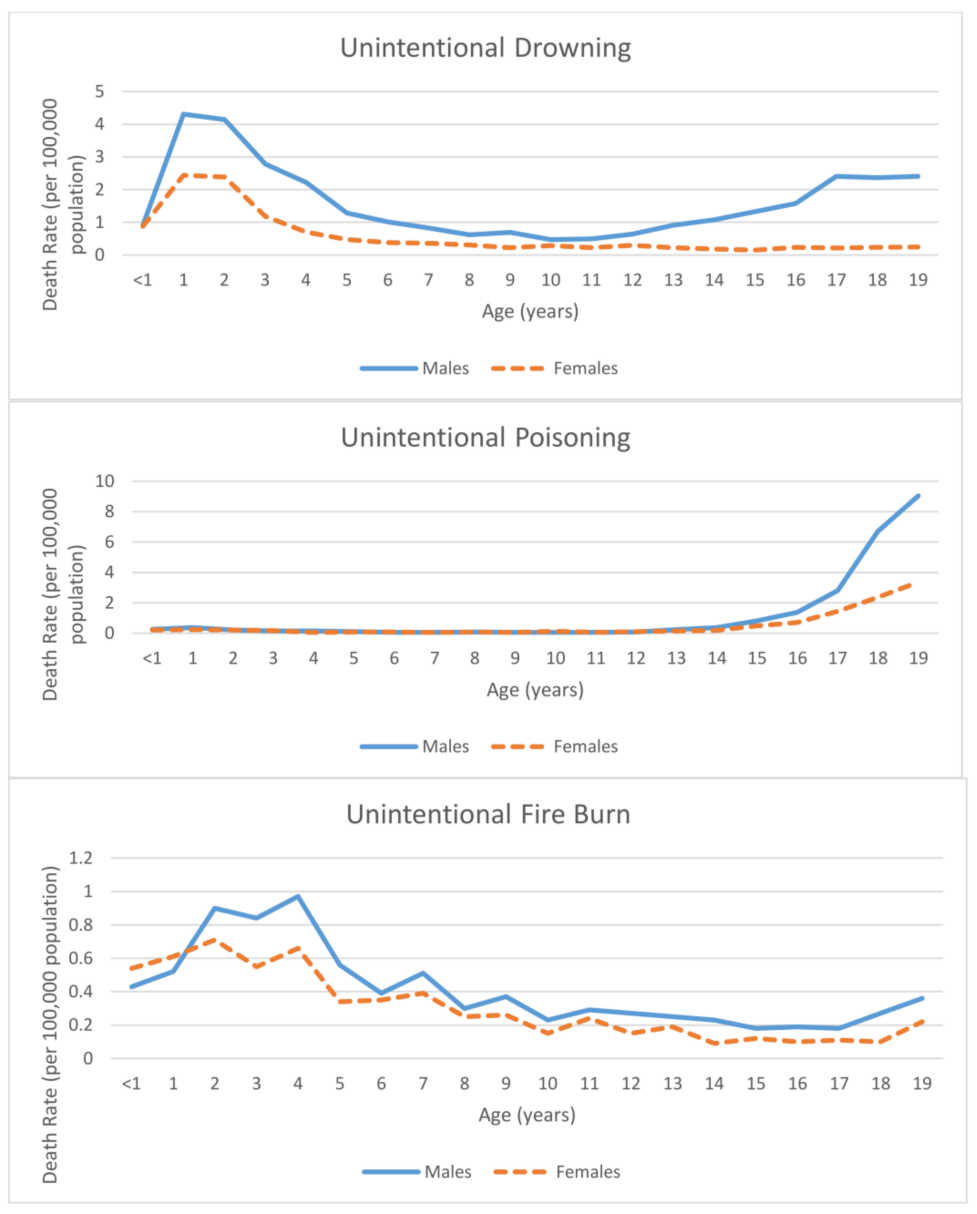

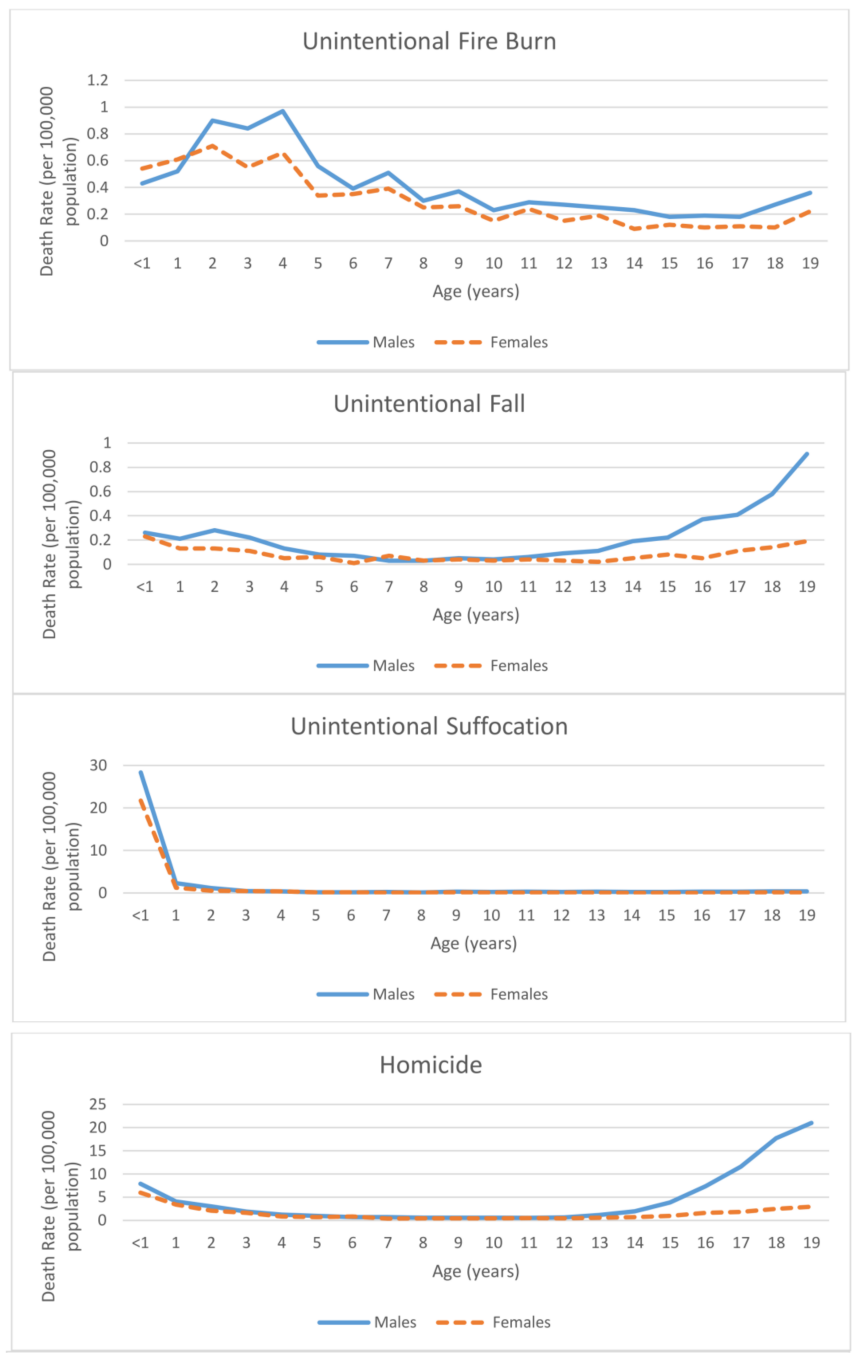

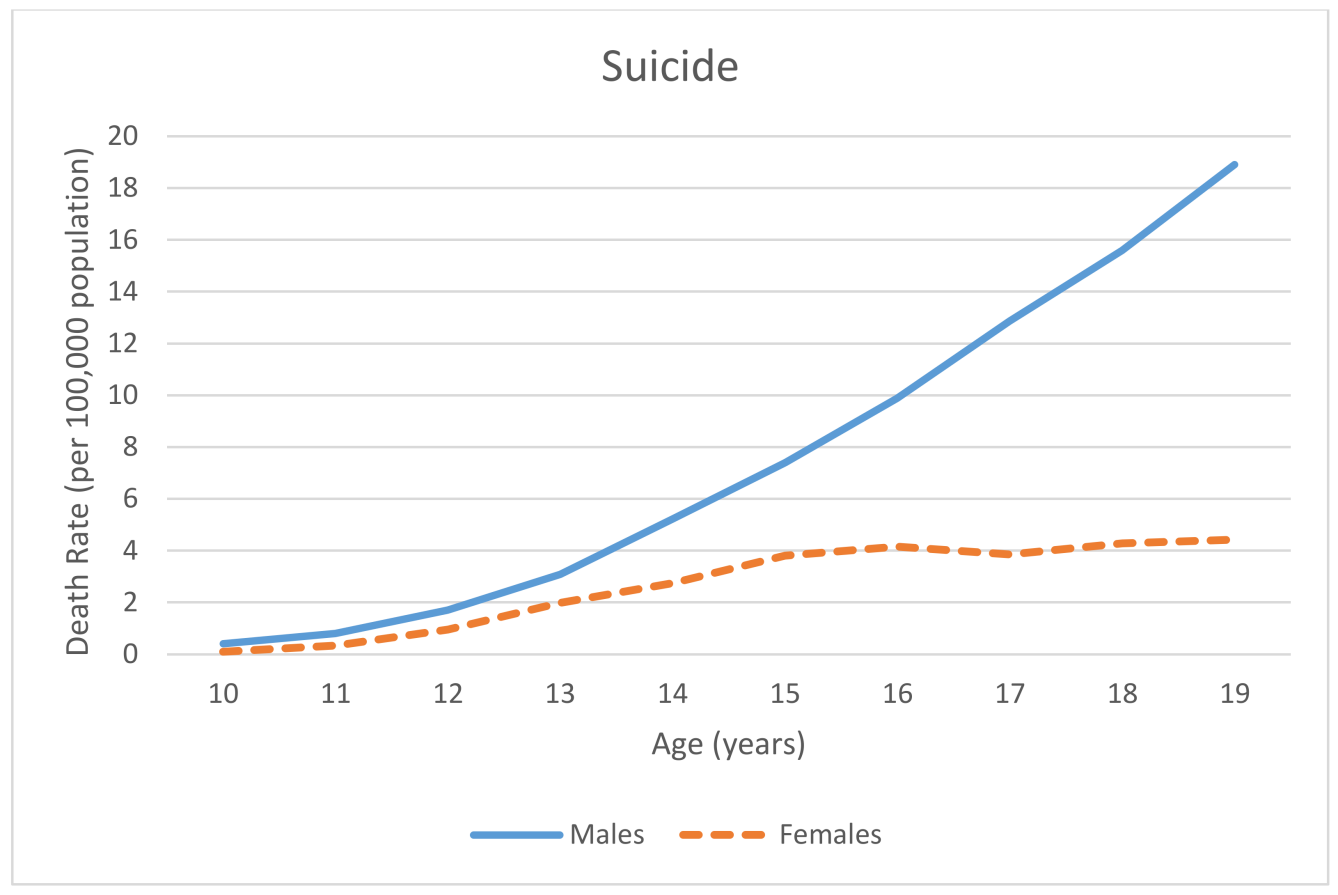
Injury and violence related death rates (per 100,000 population) among youth (ages 0–19 years) by intent, mechanism, and age group, National Vital Statistics System, United States, 2011–2015. Note: The scales for the vertical axes are different for each intent and mechanism.

The patterns of death rates widely varied when looking at specific injury intents, mechanisms, sex, and year of age as seen in Figure 5 (note that the scales for the vertical axes are different for each intent and mechanism). For example, MV occupant death rates were essentially equal for males and females through the age when adolescents begin to drive on their own; then the male rate is higher than the female rate; unintentional poisonings, homicides, and suicides had a similar age/gender patterns with the divergence between males and females widening with age. For some mechanisms, death rates substantially varied by age. For example, unintentional suffocation death rates were essentially zero for individuals older than 12 months, unintentional drowning and fire/burn death rates peaked for children at 1–4 years old. Some death rates for specific mechanisms and intents were very low, including unintentional poisonings among those less than 15, unintentional falls among 7–12 year olds, unintentional suffocations for all older than 12 months, and homicides among 7–13 year olds. 

## 4. Discussion

Several published studies have examined the epidemiology of fatal and non-fatal injuries among U.S. children and adolescents [2,8,9,10,11,12]. Our study complements previous work by using the more recent data, and presenting information on injuries from all intents (i.e., unintentional, homicide/assaults, and suicide/self-harm). The results indicate several major areas of concern, most notably the rising death rates among certain age groups for unintentional injuries and for suicide. 

As expected, the most common causes of injury death varied by age group. Infants were at most risk for suffocations, while those 1–4 years of age were at highest risk for drowning, homicides, and MV traffic. The top three leading causes of death by age group are: ages 5–9 years, MV traffic, homicide, and drowning; ages 10–14 years, MV traffic, suicide, and homicide; and ages 15–19 years, MV traffic, suicide, and homicide. Our findings are consistent with other studies that suggest there is a relationship between the developmental stage of children and the injuries they sustain [13,14]. To be most effective, prevention strategies and programs should be appropriate for the developmental stage of their target audience [15].

Injuries by race/ethnicity showed important disparities, with all unintentional injuries ages 0–19 years showing the lowest rates among Asian/Pacific Islander and the highest rates among Blacks. These rates from 2013–2015 were patterned similarly when viewed by five-year age groups. The striking deaths rates for Blacks under age 1 and 15–19-year old AI/AN youth may be related to the contexts in which these children live, including limited access to resources, environmental factors, disparities in mental and physical health care access and utilization, and high rates of poverty [16]. High rates of homicide among Black youth ages 0–19 years old may reflect disproportionate exposure to social and environmental risk factors for violence—such as poverty, racism, and neighborhood crime—as well as limited educational and occupational opportunities [17,18,19]. High suicide rates among AI/AN may reflect social and environmental circumstances, including discrimination and historical trauma, exposure to suicides of others, and limited access to health and mental health services (Figure 4) [20].

In addition to differences by age and race/ethnicity, we found that the leading causes of injury morbidity and mortality also greatly differed by sex. These results are consistent with previous child injury epidemiology conducted by the CDC [21]. Some of these differences can be attributed to societal expectations, exposure to risks, and behavioral risk factors of the injured child. Injury risk is influenced by a child’s temperament (i.e., activity level, impulsivity, and inhibitory control), personality (i.e., sensation seeking), psychosocial and cognitive development (e.g., ability to appraise the risk of a situation, estimation of one’s own physical ability) [22]. Additionally, research suggests that for young children, supervision by parents and caregivers can also affect risk for unintentional injury [23]. Supervision can be a powerful protective factor to prevent unintentional injuries, especially for infants and toddlers. Dimensions of supervision include attention (e.g., engagement and interaction with child), proximity (e.g., physical closeness, touching, within reach), and continuity (e.g., continuous, intermittent, or absent) [24].

The field of violence prevention has learned a great deal about which programs, policies, and practices are effective at reducing violence or key risk factors for violence. To help states and communities make decisions about prevention options, the CDC has released a series of technical packages that describe the best available evidence. The technical packages cover suicide, child abuse and neglect (data not specifically presented in this analysis), youth violence, sexual violence, teen dating violence, and intimate partner violence prevention [25,26,27,28,29]. The strategies span the social ecology to reduce risk and enhance protection at the individual, relationship, community, and societal levels. Strategies aim to support safe, stable, nurturing relationships and environments by ensuring a strong start through early childhood education, teaching problem solving and relationship skills, promoting social norms that protect against violence, engaging influential adults and peers, creating protective environments, strengthening economic and other supports to families, and providing support to lessen harms and prevent future risk. 

In unintentional injury prevention, implementing effective prevention strategies, such as using seat belts and child safety seats; disposing of unused, unneeded, or expired prescription drugs; wearing bicycle and motorcycle helmets; installing residential smoke alarms; reducing the use of alcohol (data not specifically presented in this analysis); using life jackets; installing four-sided self-latching pool fencing; strengthening graduated driver licensing laws; using safety equipment and implementing injury prevention strategies in sports (data not specifically presented in this analysis); and protecting young workers from injuries on the job will all contribute to reducing unintentional injuries. The CDC National Action Plan for Child Injury Prevention, developed by CDC and more than 60 stakeholder organizations to spark coordinated action to prevent unintentional injuries to children and youth [30], provides a roadmap for strengthening the collection and interpretation of data and surveillance, promoting research, enhancing communications, improving education and training, advancing health systems and health care, and strengthening policy. 

To address the prevention of motor vehicle crash injuries, the leading cause of unintentional injury deaths among those aged 5–19 years, the World Health Organization, in collaboration with global partners including CDC, has produced a technical package on traffic injury prevention. The Save LIVES: A Road Safety Technical Package [31] is an evidence-based inventory of priority interventions with a focus on speed management, leadership, infrastructure design and improvement, vehicle safety standards, enforcement of traffic laws, and post-crash survival. The package prioritizes these 6 strategies and details 22 interventions addressing key risk factors, as well as providing guidance on their implementation to save lives on the road. This road safety policy package can help guide decision makers on reducing motor vehicle injuries among children and youth. WHO also produced a World Report on Child Injury Prevention [32], which is a roadmap to keep kids safe by promoting evidence-based injury prevention interventions across all unintentional injury types, with a focus on the five most important causes of unintentional injury—road traffic injuries, drowning, burns, falls, and poisoning—and includes seven recommendations for action. This analysis presents the U.S. burden for these main causes.

Our study is subject to several limitations. First, it was not possible to examine injury deaths by state and by year, because the low number of deaths in some states would have resulted the need to suppress results to protect the privacy of decedents. Therefore, we used the most recent seven-year data window to show some state differences. Examining deaths at the county-level, which could better inform local decisions on prevention, would pose similar challenges. Second, some death rates presented in this paper are considered unstable because based on a small number of deaths. Third, NEISS-AIP data only include individuals who are seen in an emergency department, so injuries treated in doctors’ office, in urgent care centers, or left untreated are not included. For example, many young people are victims of violence, but are not treated in emergency departments. Injury is one aspect of the burden of violence, but it is important to remember that many incidents of violence, such as bullying, neglect, and dating violence or sexual abuse may not result in physical injuries, but can have significant and lasting negative consequences for victims. Fourth, NEISS-AIP is designed to give only national estimates, so state-level analyses were not possible. Fourth, information on race and ethnicity are not collected consistently in the NEISS-AIP because data are from medical records from emergency departments. Fifth, we did not present national estimates using the NEISS-AIP by injury cause, intent, and single year of age, because most of these rates were unstable due to small case counts. Lastly, the data used here may underreport races other than white and black, and underreport Hispanic origin [33].

## 5. Conclusions

Almost 13,000 children and adolescents died in 2015 from unintentional or violence-related injury. This is a substantially smaller number than the 17,500 people who died in 1999 [1] from injuries or violence, an indication that childhood injury prevention efforts are succeeding; however, the rising death rates among certain age groups, races, and in specific geographical locations for unintentional injuries, homicide, and suicide is a concern.

Our results indicate that, in an effort to reduce violence and unintentional injuries to children, it is important to consider cause, intent, age, gender, race, and regional factors to assure that prevention resources are directed to those at greatest risk. Prevention opportunities extend beyond addressing individual risk behaviors to include programs, policies, and strategies that enhance protective factors within the family, schools, neighborhood, and community. The frequency, severity, and potential for death and disability of these injuries together with the high success potential for prevention, make injury prevention a key public health goal to improve child and adolescent health in the future. 

## Figures and Tables

**Table 1:** Fatal and non-fatal injuries among youth aged 0–19 years by intent, sex, and age group, National Vital Statistics System and National Electronic Injury Surveillance System-All Injury Program, United States, 2015.

	Deaths			Non-Fatal			
	Number	%	Rate	Number	95% CI	%	Rate
Intent							
Unintentional	7963	61.4%	9.7	7,680,704	6,413,696–8,97,712	94.7%	9352.4
Homicide */Assault *	2544	19.6%	3.1	305,183	223,016–387,349	3.8%	371.6
Suicide/Self Harm	2470	19.0%	3.0	129,645	105,176–155,977	1.6%	157.9
Sex							
Male	8951	69.0%	21.3	4,610,917	3,861,281–5,362,064	56.9%	10,990.2
Female	4026	31.0%	10.0	3,504,615	2,942,641–4,066,941	43.2%	8724.3
Age Group							
<1	1554	12.0%	39.1	223,797	167,767–279,828	2.8%	5625.8
1–4	1604	12.4%	10.1	1,783,392	1,419,085–2,147,715	22.0%	11,195.7
5–9	895	6.9%	4.4	1,620,968	1,345,861–1,897,923	20.0%	7912.1
10–14	1330	10.2%	6.4	1,964,533	1,665,919–2,263,147	24.2%	9526.2
15–19	7594	58.5%	36.0	2,522,842	2,145,646–2,900,036	31.1%	11,951.6
Total	12,977	100.0%	16.2	8,115,532	6,807,635–9,425,292	100.0%	9881.8

* includes legal intervention and terrorism, but excludes operations of war.

**Table 2:** Leading causes of injury and violence related deaths among youth aged 0–19 years, National Vital Statistics System, United States, 2015.

<1 Years		1–4 Years		5–9 Years		10–14 Years		15–19 Years	
	Number		Number		Number		Number		Number
Unintentional	1291	Unintentional	1235	Unintentional	755	Unintentional	763	Unintentional	3919
Suffocation	1125	Drowning	390	MV Traffic	351	MV Traffic	412	MV Traffic	2535
MV Traffic	64	MV Traffic	332	Drowning	129	Drowning	87	Poisoning	676
Drowning	30	Suffocation	131	Fire/Burn	72	Other Land Transp.	51	Drowning	225
Fire/Burn	22	Fire/Burn	100	Other Land Transp.	32	Fire/Burn	41	Other Land Transp.	85
Nat/Environment	12	Pedestrian	75	Suffocation	31	Poisoning	36	Fall	64
Poisoning	9	Fall	30	Nat/Environment	24	Suffocation	26	Firearm	52
Other Land Transp.	5	Poisoning	29	Pedestrian	20	Firearm	15	Pedestrian	51
Struck by/Against	5	Struck by/Against	27	Poisoning	17	Other Transp.	14	Suffocation	32
Fall	4	Firearm	25	Struck by/Against	17	Fall	13	Fire/Burn	26
Pedestrian	2	Nat/Environment	25	Fall	12	Pedestrian	11	Other Transp.	22
Homicides *	263	Homicides *	369	Homicides *	140	Homicides *	158	Homicides *	1614
Suffocation	24	Firearm	50	Firearm	69	Firearm	121	Firearm	1422
Drowning	8	Suffocation	31	Cut/Pierce	11	Cut/Pierce	8	Cut/Pierce	99
Firearm	8	Drowning	14	Suffocation	10	Fire/Burn	7	Suffocation	14
						Suicides	409	Suicides	2061
						Suffocation	234	Firearm	877
						Firearm	139	Suffocation	847
						Poisoning	23	Poisoning	148

Transp = transportation; Nat = natural. * Includes legal intervention and terrorism, but excludes operations of war.

**Table 3:** Leading causes of non-fatal injuries among youth aged 0–19 years, National Electronic Injury Surveillance System-All Injury Program, United States, 2015.

<1 Years		1–4 Years		5–9 Years		10–14 Years		15–19 Years	
	Number		Number		Number		Number		Number
Unintentional	220,498	Unintentional	1,761,604	Unintentional	1,598,395	Unintentional	1,865,190	Unintentional	2,235,017
Fall	121,531	Fall	770,250	Fall	617,362	Fall	528,190	Struck By/Against	493,123
Struck By/Against	27,829	Struck By/Against	295,509	Struck By/Against	362,955	Struck By/Against	516,099	Fall	398,076
Other Bite/Sting	12,987	Other Bite/Sting	157,851	Other Bite/Sting	102,571	Overexertion	273,389	Overexertion	333,062
Foreign Body	8181	Foreign Body	119,900	Cut/Pierce	96,322	Cut/Pierce	104,152	MV-Occupant	261,636
Fire/Burn	7882	Cut/Pierce	73,358	Overexertion	79,715	MV-Occupant	75,145	Cut/Pierce	158,692
Inhal/Suffocation	7674	Overexertion	61,032	Pedal Cyclist	61,477	Pedal Cyclist	67,167	Poisoning	80,971
Cut/Pierce	5601	Fire/Burn	44,985	MV-Occupant	61,222	Other Bite/Sting	63,771	Other Bite/Sting	73,611
Overexertion	4433	Dog Bite	32,425	Foreign Body	57,911	Other Transport	42,389	Other Transport	52,348
Poisoning	4153	Poisoning	30,767	Dog Bite	34,955	Dog Bite	27,675	Pedal Cyclist	50,144
MV-Occupant	2568	MV-Occupant	26,638	Other Transport	32,854	Foreign Body	22,610	Fire/Burn	26,826
Assault—All Causes *	3299	Assault—All Causes *	21,788	Assault—All Causes *	22,573	Assault—All Causes *	62,606	Assault—All Causes *	194,916
Struck By/Against	2460	Sexual Assault	10,333	Struck By/Against	13,267	Struck By/Against	49,363	Struck By/Against	142,628
Poisoning	189	Struck By/Against	8390	Sexual Assault	7252	Sexual Assault	6735	Sexual Assault	15,377
Fall	185	Fire/Burn	990	Other Bite/Sting	760	Cut/Pierce	1602	Cut/Pierce	12,297
						Self-harm—All Causes	36,737	Self-harm—All Causes	92,908
						Poisoning	14,267	Poisoning	42,632
						Cut/Pierce	12,799	Cut/Pierce	28,087
						Struck By/Against	1849	Struck By/Against	3601

Unk = unknown; MV = motor vehicle; Inhal = inhalation. * Includes legal intervention.
